# Effect of silver and graphene nanoparticles on the thermophysical performance of ethylene glycol-glycerol hybrid nanofluids

**DOI:** 10.1371/journal.pone.0335613

**Published:** 2025-11-04

**Authors:** Athirah Najwa Zaaba, Ali Samer Muhsan, Mohammad Shakir Nasif, Muhammad Umair Shahid

**Affiliations:** 1 Mechanical Engineering Department, Universiti Teknologi PETRONAS, Perak, Malaysia; 2 Center of Innovative Nanostructures and Nanodevices, Universiti Teknologi PETRONAS, Perak, Malaysia; Xi'an Jiaotong University, CHINA

## Abstract

This study examines the thermophysical properties of ethylene glycol–glycerol (60:40 v/v) hybrid nanofluids containing graphene nanoplatelets (GNPs) and silver nanoparticles (Ag) at concentrations of 0.1–0.5 vol.%. The nanofluids were synthesized using a two-step method with Tween-80 surfactant to enhance dispersion stability. High-resolution transmission electron microscopy (TEM) and Raman spectroscopy confirmed the morphology, lateral size, few-layer structure of GNPs, and the attachment of Ag nanoparticles. The addition of surfactant increased the zeta potential from 15.7 mV to 35.2 mV for the 0.1 vol.% GNPs/Ag formulation, indicating a marked improvement in colloidal stability. Thermal conductivity enhancement reached 102.85% at 0.1 vol.% with only a 19.84% viscosity increase. Higher nanoparticle loadings improved conductivity further but caused significant viscosity increases and reduced stability. Specific heat capacity decreased by up to 46.45%, potentially benefiting rapid thermal response applications but limiting heat storage capacity. Comparison with recent literature showed that the present formulation outperforms several similar Ag- and GNP-based nanofluids in thermal conductivity enhancement while maintaining manageable viscosity. This study is the first to report such high conductivity improvement in an EG–GLY-based hybrid nanofluid at ultra-low loading, achieved through optimized surfactant use, validated structural characterization, and benchmarking against literature. Low-concentration GNPs/Ag hybrid nanofluids, particularly at 0.1 vol.%, offer strong potential for thermal management applications where high heat transfer performance and acceptable pumping requirements are critical. However, stability limitations at higher concentrations and viscosity–conductivity trade-offs highlight the need for further optimization before large-scale deployment.

## Introduction

The rapid advancement in technology has increased the demand for high-performance cooling nanofluids, which offer superior thermal conductivity, thermal diffusivity, and convective heat transfer capabilities [1,2]. Recently, hybrid nanofluids have emerged as a promising class of heat transfer fluids, providing tunable thermophysical properties tailored to specific requirements. By incorporating one or more nano-additives, these properties can be precisely controlled to suit various applications [3,4]. The selection of appropriate base fluid is crucial for nanofluid design and preparation, as it serves as the medium for nano-additive dispersion and influences the overall thermophysical properties. Effective base fluids must exhibit thermal stability, appropriate viscosity, high heat capacity, and suitable freezing and boiling points [4–6].

Water is the most commonly used cooling base fluid in various thermal management systems. However, its low boiling and freezing points limit its application in extreme environments [7]. In severe weather conditions, such as temperatures around -20°C, mixtures of propylene glycol (PG) and water or ethylene glycol (EG) and water are often used to lower the freezing point of water. The ratio of PG/water or EG/water is optimized based on specific applications [4]. For example, Sahoo et al. [8] studied an EG/water mixture with a 60:40 ratio, which effectively lowered the freezing point without damaging heat exchanger tubing. In contrast, evacuated tube solar collectors, operating at temperatures ranging from 85 to 270°C, prefer EG due to its superior thermal stability compared to water, despite its moderate specific heat and viscosity. However, EG presents significant toxicity and environmental concerns [4,7].

Glycerol (GLY), a non-toxic and non-hazardous potential base fluid, offers higher thermal conductivity and superior boiling and freezing points compared to EG [9,10]. Therefore, a mixture of EG and GLY can provide balanced thermophysical properties, making it suitable for use in extreme environments. Despite the advantages, limited investigations have been reported on such high-viscosity fluid combinations [11]. Recent developments in hybrid nanofluids have explored advanced formulations for thermal management, including glycol-based systems incorporating metallic and carbonaceous nanoparticles [12,13], with notable progress reported in photovoltaic/thermal system applications [14,15].

Graphene nanoplatelet (GNP)-based nanofluids have shown remarkable potential in thermal management applications due to their excellent thermal conductivity [16,17]. Composed of over 10 graphene layers, GNPs retain many properties of single-layer graphene and are preferred over other graphene types and carbon nanotubes (CNTs) due to their low cost, ease of synthesis, and dispersion [18,19]. Numerous studies have demonstrated that graphene-based nanofluids significantly enhance the thermal conductivity of base fluids across various thermal management applications [20–26]. For instance, Yu et al. [27] observed an 86% increase in thermal conductivity of an EG base fluid with 5 vol% GNPs, and Sari et al. achieved a 24.18% increase in thermal conductivity over the base fluid (water). Moreover, Moh et al. [28] employed GNPs in thermal flat solar panels, achieving an impressive efficiency of 42%.

However, GNPs are inherently unstable in base fluids over prolonged periods. To address this, various approaches, such as GNP functionalization and the use of covalent, ionic, and non-ionic surfactants, have been reported [18,29,30]. Nonetheless, these surfactants often reduce the overall thermal conductivity of the base fluids, thereby compromising the thermal conduction potential of the GNPs in exchange for improved stability. The compromised thermal conductivity of graphene nanoplatelet (GNP)-based nanofluids, due to the use of surfactants or surface treatments, has been addressed by incorporating additional nano-additives to create hybrid nanofluids. Studies on graphene-based hybrid nanofluids, such as GNPs/BN/CNTs [31], GNPs/CNTs [32,33], GNPs/Cu [1], GNPs/Fe_2_O_3_ [34], GNPs/Al_2_O_3_ [35,36], GNPs/Pt [37], and GNPs/Ag [38], have demonstrated significant improvements in the thermal conductivity of base fluids. These improvements depend on the base fluid, the type of surfactant, and the nature of the nano-additives.

Although carbon nanotubes (CNTs) exhibit high thermal conductivity, they are prone to clustering and are challenging to stabilize [19]. Metal nanoparticles, on the other hand, offer intrinsic excellent thermal conductivities, with silver (Ag) nanoparticles having the highest thermal conductivity among them [39]. Therefore, hybrid nanofluids based on Ag and GNPs hold considerable potential for further investigation, especially with novel combinations of base fluids, such as ethylene glycol (EG) and glycerol (GLY). Recent research by Borode and colleagues [33] has identified Tween-80 (T-80) as the surfactant of choice for optimizing the properties of GNP-based nanofluids. These two nanomaterials were selected over other candidates due to their exceptional intrinsic thermal properties, documented synergistic performance in hybrid nanofluids, and proven chemical stability in ethylene glycol–glycerol mixtures. Therefore, the primary objective of this study is to systematically evaluate the thermophysical properties: thermal conductivity, viscosity, specific heat capacity, and stability of ethylene glycol–glycerol (60:40 v/v) based hybrid nanofluids containing graphene nanoplatelets and silver nanoparticles at low concentrations (0.1–0.5 vol.%). The work seeks to determine the optimal formulation that maximizes thermal conductivity while maintaining acceptable viscosity and stability, supported by detailed microstructural characterization (TEM, Raman) and direct comparison with recent literature.

## Methodology

### 1.1. Materials

Glycerol (GLY) 99% and ethylene glycol (EG) 99.5% procured from R&M Chemicals, Malaysia. Graphene nanoplatelets (GNPs) xGnP^®^ graphene nanoplatelets-grade C-750, surface area 750 m^2^/g Sigma-Aldrich, Silver (Ag) nano-powder, <100 nm particle size, contains PVP as dispersant, 99.5% trace metals basis Sigma Aldrich. All chemicals and reagents were used as received without any further purification.

### 1.2. Synthesis of GNPs/Ag nanofluid

The base fluid was prepared by mixing ethylene glycol (EG) and glycerol (GLY) in a 60:40 ratio. Prior to the addition of nanoparticles, the surfactant Tween-80 (T-80) was introduced to the prepared base solution to achieve a homogeneous and stable dispersion. Subsequently, the required amounts of GNPs and Ag nanoparticles were added to create hybrid nanofluids with various volume concentrations (0.1%, 0.2%, 0.3%, 0.4%, and 0.5%). The surfactant-to-nanoparticle ratio was maintained at 1:2. The exact formulation details, including the calculated masses and volumes of each component for all tested concentrations, are summarized in Table 1. The dispersion was subjected to probe ultrasonication (QSonica, 60% amplitude, 10s on/10s off pulse cycle, 2 h) under an ice bath to achieve homogeneous mixing and improve stability.

**Table 1:** Formulation details for hybrid GNP–Ag nanofluids.

Target vol% (total NPs)	Final batch volume (mL)	Base fluid total (mL)	EG 60% (mL)	GLY 40% (mL)	GNP volume (ÂµL)	GNP mass (mg)	Ag volume (ÂµL)	Ag mass (mg)	T-80 mass (mg)
0.1	100	99.9	59.94	39.96	50	110	50	525	317
0.2	100	99.8	59.88	39.92	100	220	100	1049	635
0.3	100	99.7	59.82	39.88	150	330	150	1574	952
0.4	100	99.6	59.76	39.84	200	440	200	2098	1269
0.5	100	99.5	59.7	39.8	250	550	250	2622	1586

### 1.3. Measurements and characterization

#### 1.3.1. Structural characterization.

The structure of graphene was characterized using a Raman spectrometer (Horiba Jobin Yvon HR800) equipped with a 514 nm laser light and a 100X lens.

#### 1.3.2. Thermal conductivity.

Thermal conductivity was measured using a commercially available portable digital thermal property analyzer, KD2 Pro (Decagon Devices, Inc., USA). This device operates on the principle of heat energy transfer and the transient line heat source principle. It consists of a single probe needle KS-1 (1.3 mm diameter x 60 mm length) with an integrated heating element, a thermo-resistor, and a microprocessor. The instrument is designed to measure thermal conductivity over a temperature range of -50 to 150°C with an accuracy of ±5%. Measurements were conducted at five different temperatures (40, 50, 60, 70, and 80°C). During the measurement process, the KS-1 sensor was fully immersed in the fluid. To prevent convection, care was taken to ensure the fluid and the surrounding area remained free from motion or vibration.

#### 1.3.3. Viscosity.

Viscosity measurements were performed at various temperatures using an Anton Paar rheometer. According to the product specifications, the measurement accuracy was 1.0% across a temperature range of −233.15 to 473.15 K. Viscosity for each sample was assessed between 313K and 353K. These viscosity values are essential for determining the heat transfer characteristics of the nanofluids.

#### 1.3.4. Density.

The densities of the prepared nanofluids were measured experimentally using a densitometer (DMA TM-1001, Anton Paar) with a measurement precision of 0.0001 g.

#### 1.3.5. Specific Heat Capacity.

Differential Scanning Calorimetry (DSC) was employed to measure the specific heat capacity of the prepared nanofluids. This destructive thermal analysis technique can be applied to both solid and liquid samples. Measurements were carried out in a 40 μl aluminum crucible, within a temperature range of 313 to 353K, and a heating rate of 10 °C/min in a purified nitrogen atmosphere. Temperature repeatability and calorimeter accuracy were 0.1 K and 1%, respectively. In this work, the thermophysical properties of the GNP/Ag hybrid nanofluids were experimentally investigated, focusing on the effects of both volume concentration and temperature.

## Results and discussion

### 1.4. Physiochemical properties analysis of nano-additives

**Fig 1:**
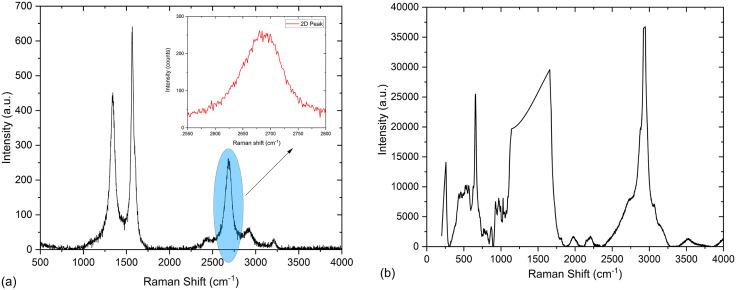
Raman spectra (a) GNPs, and (b) Ag nanoparticles.

Raman spectroscopy serves as a robust technique for probing the structural characteristics and defects of nano-additives. Fig 1(a) presents the Raman spectrum of pristine graphene nanoparticles (GNPs), revealing prominent peaks at approximately 1340 cm-1 (D), 1567 cm^-1^ (G), and 2700 cm^-1^ (2D) [40]. The D peak, associated with the breathing mode of sp^2^ carbon atoms, indicates the extent of structural defects. Comparatively, its intensity (~ 0.70, estimated via ID/IG ratio) suggests fewer defects than conventional graphene oxide flakes, typically exceeding ~0.9 [41]. The G peak, corresponding to the sp^2^ hybridized graphitic structure, exhibits sharp intensity, indicative of a regular graphitic lattice. A slight blue shift in the G peak (~1567 cm^-1^), relative to graphite (~1580 cm^-1^), suggests modification due to exfoliation. The broadened 2D peak, unlike graphite, and its splitting into multiple components indicate inter-layer interactions typical of few-layered graphene [42,43], although significant peak splitting is absent, consistent with the characteristics of GNPs.

Fig 1(b) displays the Raman spectrum of silver nanoparticles (Ag NPs), featuring a prominent peak at ~255 cm^-1^ attributed to surface vibrations [44]. Additional peaks between 430–530 cm^-1^ correspond to Ag-Ag bond stretching within nanoparticle clusters, indicative of surface plasmon resonance and a nanocrystalline structure. Two sharp peaks around ~1156 cm^-1^ and ~1654 cm^-1^ arise from vibrational modes within the bulk of the nanoparticles [45,46].

**Fig 2:**
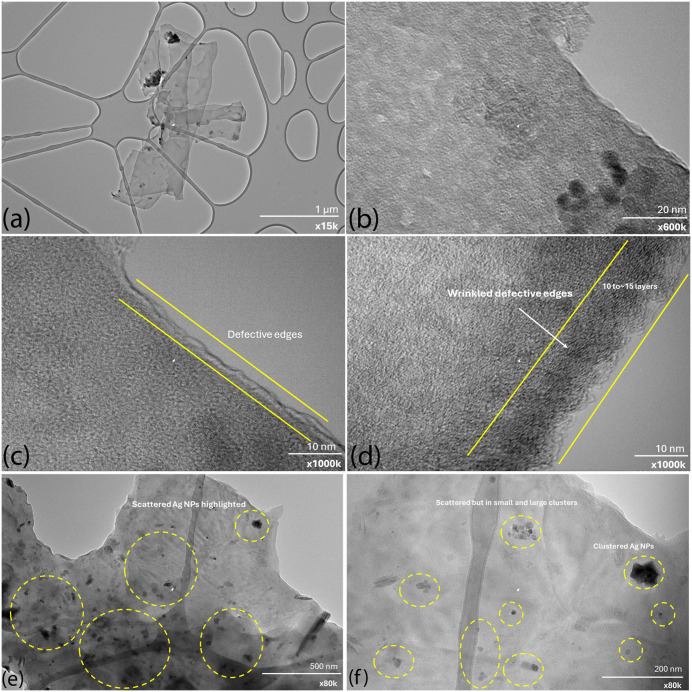
TEM images of GNPs with different magnification (a-d), and (e-f) are of GNPs/Ag hybrid particles.

TEM is a well-established analytical technique for probing the internal architecture of nanostructures [47]. It was employed to corroborate the Raman spectroscopy results and to gain deeper insight into the morphology and structure of the graphene nanoplatelets (GNPs) and their hybrids. Fig 2(a–d) present the TEM micrographs of GNPs at varying magnifications. In Fig 2(a), a cluster of graphene flakes can be observed, including a large, folded flake. The estimated lateral size of the flakes is approximately 1–2 µm, which is in good agreement with the manufacturer’s specifications. Fig 2(b) provides a magnified view focusing on the flake edges, revealing wrinkled and irregular edges, indicative of structural imperfections.

Further magnifications in Fig 2(c) and 2(d) show that the edges are highly wrinkled and defective, with no sharply defined lines. Due to the irregularity, the exact number of layers is difficult to determine; however, from the contrast and edge morphology, the flakes appear to consist of approximately 10–15 layers. The observed defective edges and estimated layer number are consistent with the Raman results, confirming the presence of few-layer, defect-rich GNPs.

Fig 2(e) shows the images of GNPs and Ag hybrid; Ag NPs are scattered over GNPs giving an impression like a dust. Fig 2(f) shows further magnified image showing that Ag NPs are scattered over the flake in clusters. Small and large clusters are clearly visible. Clustering might occur due to drawing of nanofluids as Ag NPs have very strong van der Wall interaction.

In summary, the structural observations from Raman spectroscopy and TEM analysis are in good agreement with the supplier’s technical specifications. The measured lateral dimensions from TEM (approximately 1–2 µm) and the wrinkled, irregular flake edges are consistent with a few-layer morphology containing defect sites, as also indicated by the ID/IG ratio of ~0.70 in the Raman spectrum. The broadened 2D band without sharp splitting further confirms the presence of multi-layer GNPs rather than monolayer graphene, while the observed defect density is lower than that of graphene oxide, aligning with the defect-rich but conductive nature of this grade. These correlations collectively substantiate that the carbonaceous component employed in this study meets the structural criteria for graphene nanoplatelets as defined by the manufacturer.

#### 1.4.1. Stability of GNPs/Ag hybrid nanofluid.

**Fig 3:**
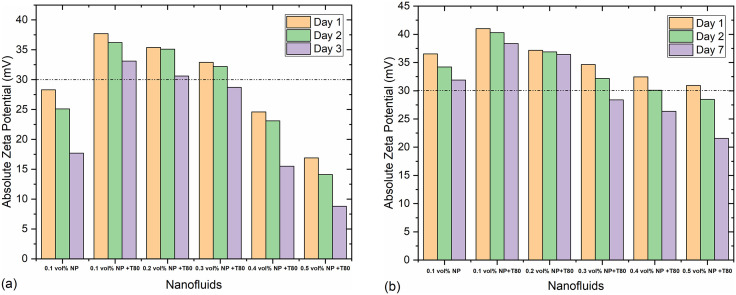
Zeta potential variation with the function of concentration of nanofluids (a) Ag-based nanofluid (b) GNPs based nanofluid.

The stability of the prepared nanofluids was assessed by monitoring zeta potential values on the first, second, and seventh days after preparation. Zeta potential reflects the surface charge of particles, crucial for understanding colloidal stability. Figure 3 presents the zeta potentials of Ag and GNP-based nanofluids across varying concentrations of nano-additives. According to the stability criterion, nanofluids are considered stable if their zeta potential exceeds ±30 mV [40].

Fig 3(a) illustrates that Ag nanoparticle-based nanofluids without surfactant (T-80) exhibit instability, as indicated by zeta potential values below 30 mV. The introduction of surfactants significantly shifts zeta potential values into the positive range, effectively enhancing stability, albeit most effective at lower concentrations (up to 0.2 vol%). A decrease of approximately 4.88% in zeta potential from day one to day seven suggests sustained stability of these nanofluids over time.

Similarly, Fig 3(b) shows zeta potential values for GNP-based nanofluids over the same period. These nanofluids, even without surfactant, demonstrate relatively higher zeta potential values, indicating better inherent stability compared to Ag nanoparticle-based nanofluids. The higher zeta potential of GNPs is attributed to their larger surface area. Minimal decreases in zeta potential over days two and seven affirm the robust stability of these nanofluids, particularly at 0.2 vol% concentration.

**Fig 4:**
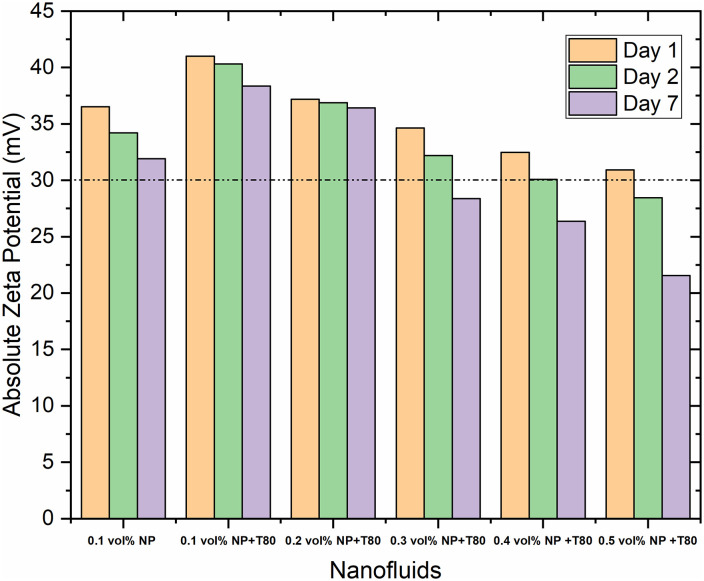
Zeta potential variations with the different concentrations of GNPs/Ag P (50:50)/EG-GLY hybrid nanofluid.

Fig 4 depicts the zeta potential values of GNPs/Ag hybrid nanofluids. Nanofluids without surfactant exhibit instability across all concentrations, with zeta potential declining as nano-additive concentration increases. Only nanofluids at 0.1 vol% concentration maintain stability, although zeta potential values approach critical thresholds upon increasing concentrations. Despite a decrease of approximately 7.14% from day one to day seven, the nanofluid at 0.1 vol.% remains reasonably stable, whereas higher concentrations fall below the stability threshold.

In summary, GNP-based nanofluids exhibit superior stability compared to Ag nanoparticle-based nanofluids, particularly evident without surfactant. The hybrid GNPs/Ag nanofluids achieve stability only at lower concentrations, emphasizing the critical role of surfactants in enhancing colloidal stability.

**Fig 5:**
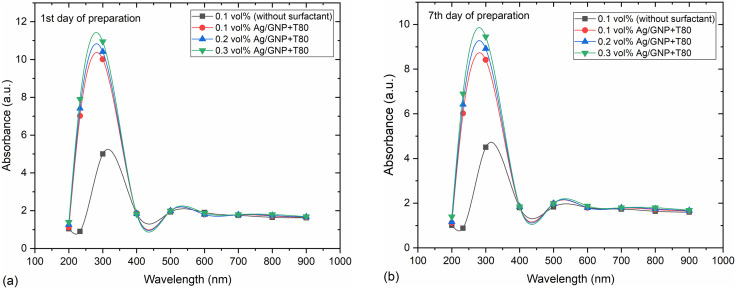
UV–Vis absorption spectra of Ag/GNP hybrid nanofluids at different concentrations: (a) day 1 and (b) day 7 after preparation.

Further analysis of the stability of GNPs/Ag hybrid nanofluids was conducted using UV-Vis spectrophotometry. Fig 5 presents the UV-visible spectra of Ag/GNP hybrid nanofluids measured on day one and after one week of preparation. A broadened peak observed at ~500–560 nm corresponds to the surface plasmon resonance (SPR) of Ag nanoparticles. The redshift and increased bandwidth may be attributed to the presence of GNPs, which modify the local dielectric environment and induce plasmon damping [48,49]. Comparison of the absorbance values between these time points reveals a minor decrease of approximately 1 absorbance unit (a.u.), indicating satisfactory stability of the nanofluids. Importantly, no shifts or changes in the symmetry of the spectra were observed over time.

**Fig 6:**
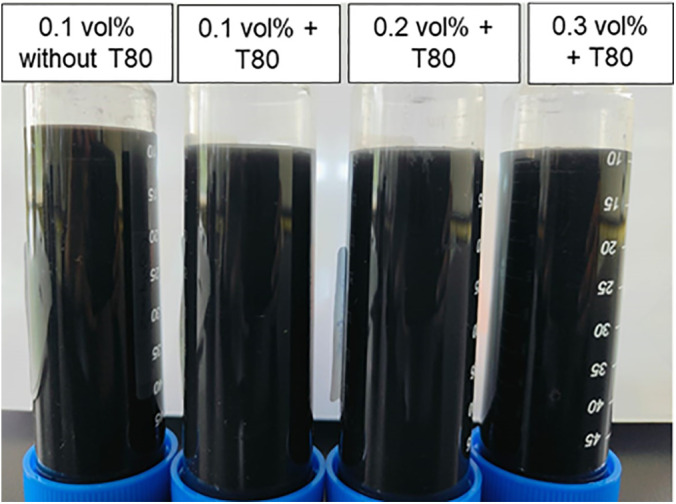
Photographs of the hybrid nanofluids 7 days after preparation.

Visual inspection, as depicted in Fig 6, corroborates these findings, showing no significant clustering of nanoparticles after one week. The UV-Vis spectrophotometer results align with this observation, confirming the stability of the hybrid nanofluids.

### 1.5. Rheological and thermophysical properties

#### 1.5.1. Thermal conductivity of base fluid and nanofluid.

**Fig 7:**
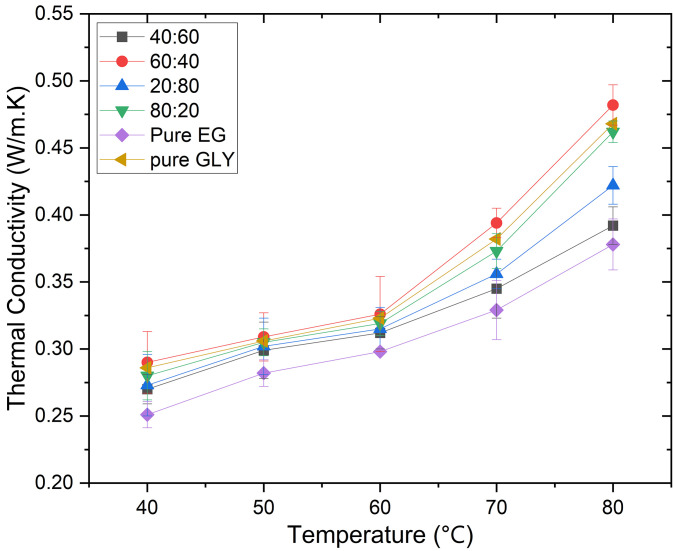
The variation of thermal conductivity of EG and GLY at different ratios.

The thermal conductivity of nanofluids is significantly influenced by the choice and composition of the base fluid. In this study, the thermal conductivity of a proposed base fluid composed of ethylene glycol (EG) and glycerol (GLY) at various mixing ratios was investigated over a temperature range of 40–80°C, as depicted in Fig 7. Increasing the temperature was observed to enhance the thermal conductivities of the base fluid, attributed to reduced inter-particle distances with increasing volume fraction [50].

Notably, pure glycerol (GLY) exhibited higher thermal conductivity compared to pure ethylene glycol (EG), with GLY showing a 23.8% enhancement at 80°C, surpassing EG. This enhancement is attributed to the reduced Brownian motion of particles due to GLY’s higher viscosity [51]. As well as due to its strong intermolecular hydrogen bonding and molecular structure. While the absolute thermal conductivity of the EG–GLY mixtures is influenced by the base fluid composition, the addition of nanoparticles consistently produces a relative enhancement for each tested ratio. This enhancement is particularly valuable in hybrid systems, where the objective is to achieve a balance between high thermal conductivity, acceptable viscosity, and long-term stability. Under real operational conditions, such as in heat exchangers or cooling loops, this balance can translate to improved overall heat transfer performance, even if the base fluid alone has a higher absolute conductivity. Among the EG/GLY mixtures tested, the base fluid composition of (EG60:40) demonstrated the highest thermal conductivity enhancement, recording a 27.5% increase relative to the base fluids. Specifically, incorporating 60% EG resulted in a 15.54% increase in thermal conductivity at 40°C. Conversely, mixtures with higher GLY content (EG 40:60 and EG 20:80) showed thermal conductivities comparable to or slightly enhanced relative to pure EG.

Given the superior enhancement in thermal conductivity observed with the (EG60:40) mixture, this composition was selected as the optimal base fluid for subsequent nanofluid formulations containing silver (Ag), graphene nanoparticles (GNPs), and their hybrid (Ag/GNP) combinations.

**Fig 8:**
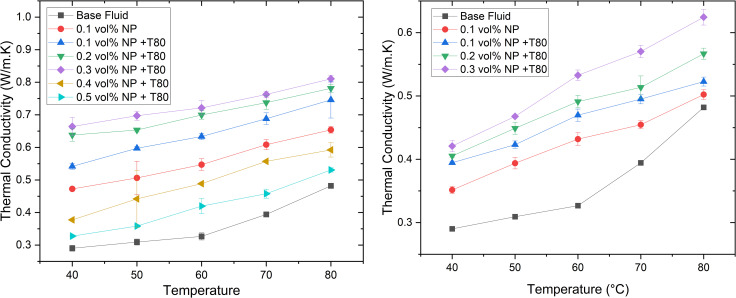
The variation of thermal conductivity of (a) Ag and (b) GNP-based nanofluids.

The thermal conductivity of silver (Ag) and graphene nanoparticles (GNPs) nanofluids in an ethylene glycol/glycerol (EG/GLY) mixture at a 60:40 ratio (by volume) was investigated across concentrations ranging from 0.1% to 0.3% vol., as shown in Fig 8(a) and 8(b) respectively. It was observed that the thermal conductivity increases proportionally with the volume fraction of nanoparticles. The optimal concentration of Ag and GNPs is influenced by surface modifications, such as surfactant addition or functional group introduction.

**Fig 9:**
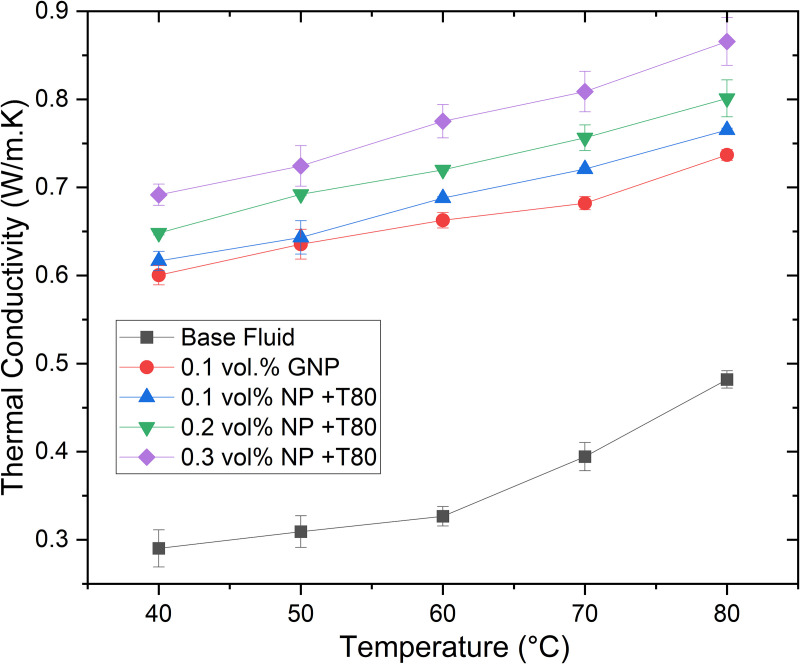
The variation of thermal conductivity of Ag/GNP-based hybrid nanofluids.

Furthermore, the thermal conductivity of hybrid nanofluids comprising Ag and GNPs in the same EG/GLY mixture ratio was studied at concentrations of 0.1% to 0.3% vol., as depicted in Fig 9. Direct comparison with base EG/GLY nanofluids was not feasible due to unavailable data. The results show a nearly linear relationship between thermal conductivity and nanoparticle volume fraction, significantly surpassing the thermal conductivity of corresponding base fluids. However, concentrations exceeding those shown for GNPs/Ag nanofluids led to particle agglomeration and settling, limiting further testing.

At an operational temperature of 60°C, the base fluid (EG/GLY) exhibited a thermal conductivity of 0.3267 W/mK. Introducing 0.1% vol. of GNPs increased thermal conductivity by 47.51%, while 0.1% vol. of Ag resulted in a 67.46% increase (0.5471 W/mK). Combining Ag with GNPs in the same nanofluid composition demonstrated even more significant enhancement, with a 102.85% increase at 0.1% vol. (0.6627 W/mK).

The enhanced thermal conductivity can be attributed to the Brownian motion of nanoparticles, which disperses more rapidly in hotter regions, thereby increasing thermal conductivity compared to the base fluid. Remarkably, the greatest improvement in thermal conductivity for GNPs/Ag-EG/GLY nanofluids was observed at lower nanoparticle concentrations (0.1% vol.), indicating substantial heat transfer enhancement while minimizing nanoparticle loading.

Further evaluation of other thermophysical properties (viscosity, density, and specific heat capacity) of hybrid nanofluids across 0.1%, 0.2%, and 0.3% vol. nanoparticle concentrations corroborated the superior thermal conductivity observed compared to single-component Ag and GNPs nanofluids.

To place the present results in context, the thermal conductivity and viscosity enhancements obtained in this study were compared with those reported in recent literature for similar nanoparticle systems and base fluids. At 0.1 vol.% loading, our Ag/GNP hybrid nanofluid in EG–GLY (60:40) achieved a thermal conductivity enhancement of 102.85% with a viscosity increase of only 19.84%. In comparison, Yarmand et al. [40] reported a 32% conductivity enhancement with a 28% viscosity increase for Ag/GNP nanofluids in pure EG at the same concentration. Borode et al. [36] observed a 45% enhancement with a 25% viscosity increase for GNP/Fe₂O₃ hybrids in EG–GLY, while Akilu et al. [4] achieved a 20.5% enhancement with a 15% viscosity increase for SiO₂–CuO/C hybrids in EG–GLY. For water-based systems, Suresh et al. [11] reported a 12% enhancement with a 22% viscosity rise for Al₂O₃–Cu hybrids. These comparisons demonstrate that the hybrid formulation used in this work delivers substantially higher thermal conductivity gains while maintaining viscosity increases within a manageable range, making it competitive for practical heat transfer applications.

#### 1.5.2. Viscosity of GNPs/Ag P hybrid nanofluid.

**Fig 10:**
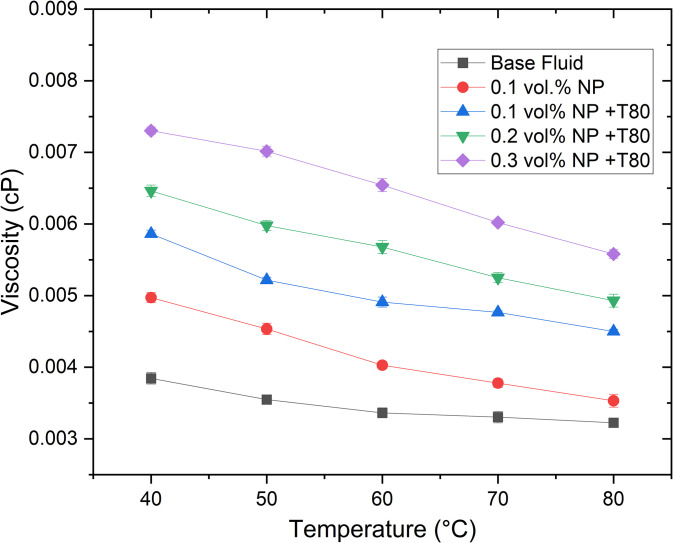
The variation of viscosity of Ag/GNPs hybrid nanofluids at 200s^−^^1^ shear rate.

The viscosity of Ag/GNP nanofluids in an ethylene glycol/glycerol (EG/GLY) mixture at a 60:40 ratio (by volume), as a function of temperature and nanoparticle volume concentration, is illustrated in Fig 10. It is evident that viscosity increases with higher nanoparticle volume concentrations. For instance, at 0.2 vol.% concentration and 60°C, viscosity increased by 68.9% compared to the base fluid. Conversely, viscosity decreases with rising temperature due to reduced intermolecular and interparticle bonding forces, as observed across the entire temperature range.

Experimental findings consistently demonstrate that nanoparticle concentration correlates directly with nanofluid viscosity, attributed to increased Van der Waals forces between base fluid molecules and nanoparticle atoms or molecules [52]. Higher concentrations of Ag/GNPs nanoparticles amplify viscosity due to increased particle collisions, as supported by prior research [53,54]. An ideal nanofluid should exhibit high thermal conductivity alongside low viscosity. However, hybrid nanofluids typically exhibit higher viscosity compared to pure base fluids. Despite this, the viscosity of nanoparticle-enriched fluids remains manageable. It is noteworthy that water, although having low viscosity, is limited in thermal system applications due to its low boiling point and susceptibility to bacterial contamination leading to corrosion [55,56]. Surfactants like Tween-80 (T80) play a significant role in reducing the viscosity of hybrid nanofluids compared to base fluids. T80 effectively mitigates nanoparticle aggregation, resulting in lower measured viscosity and substantial energy savings in pumping power at elevated operating temperatures.

The observed viscosity increase, particularly the 68.9% rise at 0.2 vol.% Ag/GNPs (Fig 10), has practical implications for flow systems. In applications such as automotive cooling loops or heat exchangers, higher viscosity can increase pumping power requirements and reduce Reynolds number, potentially shifting the flow regime from turbulent toward transitional or laminar, thereby affecting heat transfer performance.

To estimate the impact, the Darcy–Weisbach equation and the Reynolds number (Re = ρVD/μ) can be used. For example, at 0.2 vol.% loading, the viscosity increase would proportionally reduce Re by ~40% under constant flow conditions, requiring additional pumping power to maintain equivalent flow rates. This highlights the need to balance the thermal conductivity gains of hybrid nanofluids with the hydraulic penalties of increased viscosity. Based on our stability and viscosity results, concentrations ≤0.1 vol.% are recommended for long-term applications, as they offer significant conductivity enhancement while keeping viscosity increases to ~20%, which is generally acceptable in industrial cooling systems.

#### 1.5.3. Density of GNPs/Ag P hybrid nanofluid.

**Fig 11:**
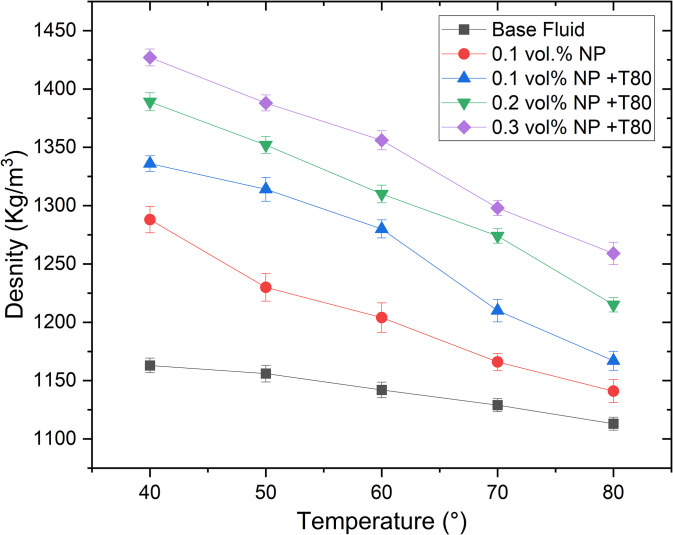
The variation of density of Ag/GNP hybrid nanofluids.

Density measurements were conducted on the ethylene glycol/glycerol (EG/GLY) base fluid at a 60:40 ratio and Ag/GNP-based hybrid nanofluids across various temperatures, with results depicted in Fig 11. It is evident that density decreases with increasing temperature for both the base fluid and hybrid nanofluid. This phenomenon is attributed to thermal expansion of the fluid as temperature rises.

At particle concentrations of 0.1%, 0.2%, and 0.3%, with Tween-80 (T80) surfactant, density increased by 14.88%, 19.43%, and 22.70%, respectively, at 40°C compared to the base fluid. Similarly, at 80°C, density rose by 4.85%, 9.16%, and 13.12% for the same concentrations, as shown in Fig 11. These findings highlight a significant increase in density with higher nanofluid volume concentrations. Consistently, the literature supports that nanofluid density increases with particle concentration [48–50], primarily due to the greater mass of nanoparticles relative to the base fluid. Conversely, the observed decrease in density with temperature is attributed to expanded intermolecular spacing as thermal energy increases.

#### 1.5.4. Specific heat (Cp) of GNPs/Ag P hybrid nanofluid.

**Fig 12:**
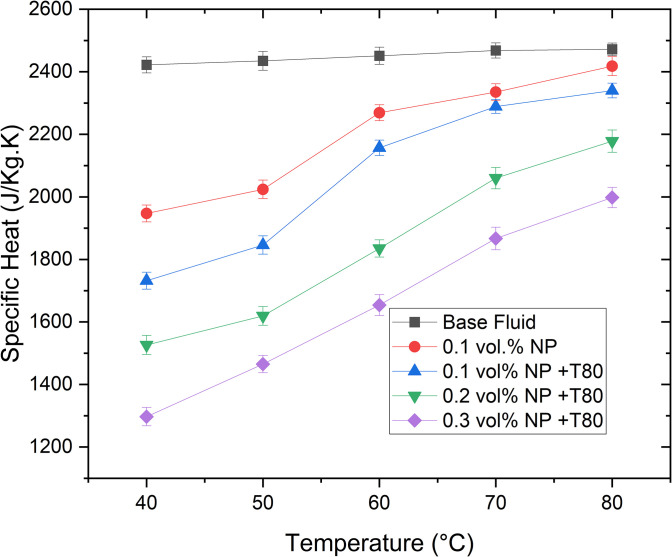
The variation of specific heat of Ag/GNPs hybrid nanofluids.

Specific heat capacity (Cp) is a critical parameter in thermal system design and performance analysis, particularly in thermal energy storage systems. Figure 12 illustrates the specific heat capacity of Ag/GNP nanofluids based on an ethylene glycol/glycerol (EG/GLY) mixture at a 60:40 ratio, as a function of temperature and nanoparticle volume concentration. It is evident that the specific heat capacity of nanofluid samples is consistently lower compared to the base fluid EG/GLY (60:40). Experimental results indicate a maximum decrease of 46.45% in specific heat capacity compared to the base fluid at 40°C. This suggests that nanofluids require less heat energy to achieve temperature increases compared to the pure EG/GLY base fluid. Furthermore, specific heat capacity decreases with increasing nanoparticle concentration. This phenomenon can be attributed to the surface free energy effects on graphene nanoparticle (GNP) specific heat capacity, influenced by their large surface area. However, specific heat capacity increases with rising temperature, which affects Cp more significantly than nanoparticle mass fraction. This behavior is typical, as increasing nanoparticle concentration or adding surfactants enhances heat transfer capabilities while reducing initial thermal storage capacity of the fluid [32,57].

This observed decrease in Cp implies that the hybrid nanofluid can heat up more rapidly under a given heat flux, which may be beneficial for applications requiring fast thermal response, such as dynamic cooling of high-performance electronics or rapid thermal cycling processes. However, the reduced Cp also diminishes the fluid’s ability to store heat, which can negatively affect applications that rely on high thermal energy storage capacity, such as concentrated solar thermal systems or load-leveling thermal reservoirs. Therefore, the choice of nanoparticle loading should be guided by the intended application, balancing thermal conductivity enhancement with the trade-off in heat storage capability.

## Conclusion

This study investigated the thermophysical properties of ethylene glycol–glycerol (60:40 v/v) hybrid nanofluids containing graphene nanoplatelets (GNPs) and silver nanoparticles (Ag) at concentrations ranging from 0.1 to 0.5 vol.%. The prepared hybrid nanofluids exhibited significant thermal conductivity enhancement, with the 0.1 vol.% formulation achieving a 102.85% increase compared to the base fluid while maintaining a moderate viscosity increase of ~19.84% and stable dispersion (|ζ|>30 mV). Higher nanoparticle loadings further improved thermal conductivity but were accompanied by substantial viscosity increases and reduced colloidal stability, limiting their suitability for long-term operation. The specific heat capacity decreased with increasing nanoparticle concentration, which can be advantageous for applications requiring rapid thermal response but may be detrimental in systems where high thermal energy storage capacity is needed. The observed viscosity–conductivity trade-off suggests that an optimal concentration exists for each application, balancing enhanced heat transfer with acceptable pumping power requirements. Overall, the results indicate that low-concentration GNPs/Ag hybrid nanofluids, particularly at 0.1 vol.%, offer strong potential for use in thermal management applications such as heat exchangers, automotive cooling, and electronics thermal regulation, where stability and manageable viscosity are essential. However, further optimization, long-term stability testing, and application-specific performance evaluations are recommended before large-scale deployment.
